# Single-pixel polarimetric direction of arrival estimation using programmable coding metasurface aperture

**DOI:** 10.1038/s41598-021-03228-5

**Published:** 2021-12-13

**Authors:** The Viet Hoang, Vincent Fusco, Muhammad Ali Babar Abbasi, Okan Yurduseven

**Affiliations:** Institute of Electronics, Communications, and Information Technology (ECIT), Queen’s University Belfast, Belfast, BT3 9DT UK

**Keywords:** Electrical and electronic engineering, Applied mathematics

## Abstract

This paper presents a single-pixel polarimetric compressive sensing (CS)-based direction of arrival (DoA) estimation technique using a cavity backed programmable coding metasurface aperture. The single-pixel DoA retrieval technique relies on a dynamically modulated waveform diversity, enabling spatially incoherent radiation masks to encode the incoming plane waves on the radar aperture using a single channel. The polarimetric nature of the wave-chaotic coded metasurface ensures that the DOA estimation is sensitive to the polarization state of the incoming waves. We show that the polarimetric single-pixel DoA concept can be realized by encoding the polarization information of the incoming waves at the physical layer level within the antenna. A dynamically reconfigurable wave-chaotic metasurface, which possesses a structured sparsity of dual-polarized coded metamaterial elements, is proposed for the proof of concept. It is shown that by encoding and compressing the source generated far-field incident waves into a single channel, we can retrieve high fidelity polarimetric DoA information from compressed measurements.

## Introduction

Polarization is an important property of wavefronts with a great potential in various application scenarios such as wireless communication^1–7^, radar^8–11^, and imaging^12–14^. Because of the reduced multipath and antenna orientation constraints, dual-polarization and circular polarization are often suitable selections in wireless communication applications^1–7^. Moreover, polarization-diversity has been leveraged in a variety of other applications in the literature. For example, target polarization properties were analyzed in remote sensing and synthetic aperture radar (SAR) applications in^8,9^, while polarimetric airborne and spaceborne radar platforms were discussed in^10,11^. In near-field imaging, polarimetric imaging can be employed to obtain additional information from the target^12–14^. In addition, polarization was shown to be incorporated in antenna arrays in radar systems in order to improve the signal parameter estimation, including direction of arrival (DoA)^15,16^.

DoA estimation is a fundamental problem in array signal processing and plays a vital role in automotive radar^17^, sonar^18^, channel sounding^19^, and wireless communications^20^. Conventional DoA estimation schemes are based on beamforming techniques, or numerical methods such as MUSIC^21,22^ and ESPRIT^23–25^, with the hardware layer relying on a dense array-based system architecture as a receiving unit. Typically, the antenna array is synthesized at the Nyquist spatial sampling of half of the operational wavelength. This traditional array-based approach makes it necessary to collect the received radar signals from multiple channels (i.e. multi-pixel architecture) and process them using sophisticated DoA estimation algorithms to retrieve the DoA information of incoming far-field sources. The drawback of the multi-pixel approach is that, as the operating frequency increases, the number of antennas (and hence the number of data acquisition channels) also increases, and for maximum accuracy, matched channel calibration is required. This can result in a rather complex system architecture at the receiver unit, especially at millimeter-wave and submillimeter-wave frequencies where radio hardware is expensive and lossy. With a higher number of antenna elements in a dense array, the associated radio frequency (RF)-chains are bound to increase, putting a significant pressure on the hardware requirement and deployment cost. As an enabling technology, the compressed sensing paradigm with a single-pixel-based metasurface aperture can substantially simplify the physical layer hardware for DoA estimation. A significant advantage of this technique is that the received data from the source is compressed into a single channel, circumventing the necessity to have array-based multiple channels to retrieve the DoA information.

Recently, DoA estimation using single-pixel-based frequency-diverse or dynamically modulated metasurface apertures has gained significant attention^26–29^. In^26,27^, the authors proved that frequency-diverse apertures can be used to retrieve the DoA information of incoming far-field sources. In this method, the characteristics of the aperture radiated fields are governed by a frequency sweep, with the aperture radiating a distinct radiation pattern at each frequency, which is ideally, orthogonal to the radiated field patterns at other discrete frequencies. The radiation of random, complex modes varying as a function of frequency can be performed by mode-mixing cavities, e.g. as presented in^26,27^. However, this technique exhibits several disadvantages, such as the necessity to sweep a rather large bandwidth, leading to complicated hardware design, and possible interference within the operating bandwidth due to the overly-congested microwave spectrum. Therefore, to overcome these drawbacks, dynamically controlled metasurfaces, such as the ones proposed by the authors in^28,29^, can be used as an alternative solution. The single-pixel programmable metasurface aperture produces tailored radiation patterns flexibly governed by dynamically tuning the radiating elements on the aperture over a narrow operating bandwidth, or even at a single frequency, thereby reducing hardware component complexity. However, despite the advantages offered by the single-pixel physical layer compression, all of these CS-based DoA estimation modalities are scalar, i.e. they lack the ability to retrieve the polarimetric nature of the incoming waves. As a result, such scalar CS-based DoA estimation techniques cannot detect the polarization state of the incoming waves. This poses a fundamental limitation because the polarization of the incoming waves on the receiver aperture can be diverse, making it necessary to have a polarimetric aperture layout for a practical CS-based DoA estimation technique.

In this paper, we bridge this gap by demonstrating that the single-pixel non-polarimetric (or scalar) CS-based DOA estimation technique can be redeveloped to exhibit a polarimetric setting by encoding the polarization information of the incoming waves in the physical layer, at the antenna level. A dynamically reconfigurable wave-chaotic metasurface antenna that possesses a structured sparsity of dual-polarized coded metamaterial radiator elements is proposed for this purpose. It is demonstrated that by encoding and compressing source-generated far-field incident waves into a single channel using spatio-temporally incoherent measurement modes generated by the coded programmable metasurface, we can retrieve high fidelity polarimetric DoA patterns from the compressed measurements. Furthermore, the sparse array of radiator elements on the metasurface aperture can reduce the hardware cost compared with a conventional dense array due to the physical layer level compression property.

## Methods

### Polarimetric DOA estimation

The first DoA estimation using a single-pixel based frequency-diverse metasurface aperture was proposed in^26^. Afterwards, similar principles were applied to estimate a far-field source distribution from samples of the coherence function multiplexed according to a linear matrix equation defined in terms of complex-valued modulation patterns and single-pixel measurements using dynamically tunable, programmable wave-chaotic metasurface aperture^28,29^. These techniques operate on a scalar approximation, ignoring the polarization content of the incoming waves generated by far-field sources. This poses a limitation, in that there is no guarantee that the metasurface apertures have a meaningful signal reception if the polarization of the incoming wave is different from the dominant polarization of the metasurface aperture.

**Figure 1:**
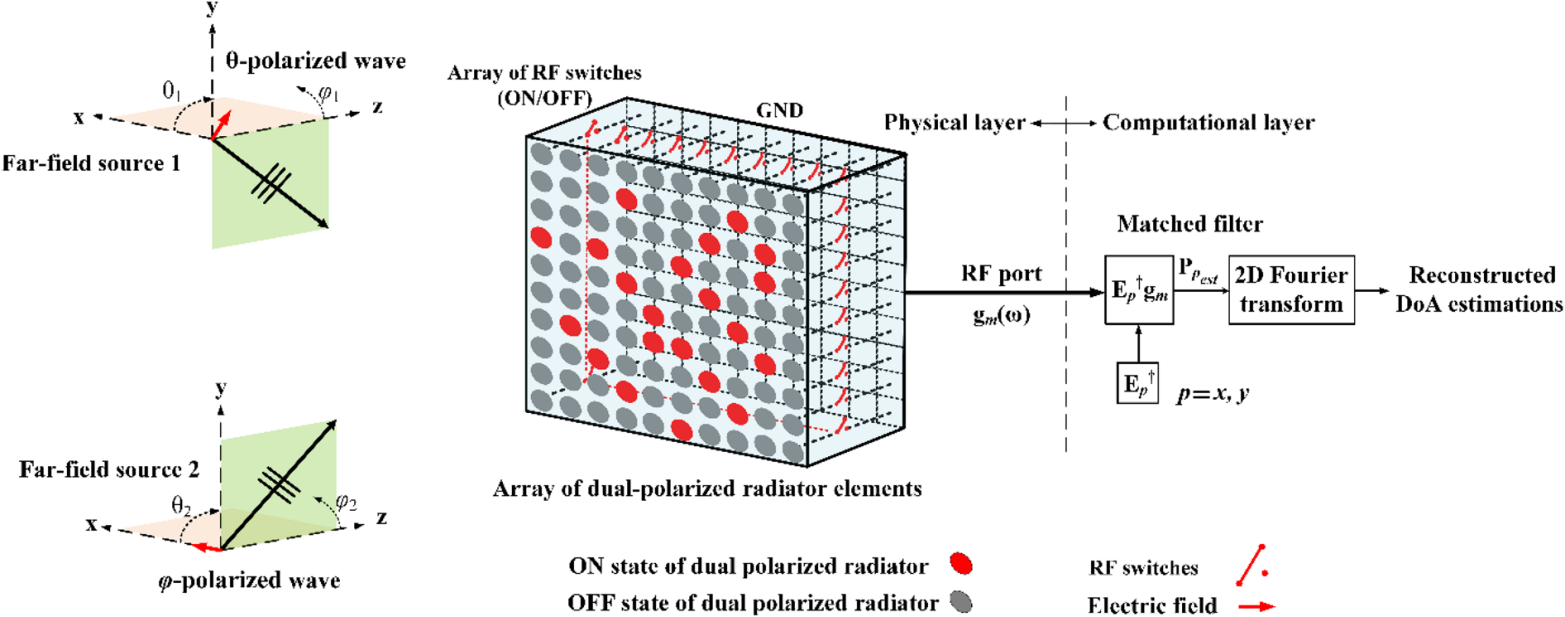
Illustration of the proposed programmable aperture for polarimetric DOA estimation concept.

In this paper, a passive, single-pixel, polarization agnostic CS concept for DoA estimation methodology illustrated in Fig. 1 is developed to react to the change of the polarization of the incoming wave using an array of dual-polarized radiating elements. In the physical layer, the radiation of the elements is controlled by an array of RF switches. When a switch is opened, the element connected with the switch radiates (i.e., *on* state) and when this switch is closed, the element connected with the switch does not radiate (i.e., *off* state). By randomly changing the *open/close* positions of the switches, the synthetic radiation pattern formed as a linear superposition of the patterns of the radiated elements is dynamically changed. All elements are connected to a single RF port in reception, and hence the whole system is seen as a single-pixel structure. DoA estimation using this single-pixel structure is achieved by capturing the projection of a far-field source on the aperture through the spatio-temporally diverse patterns of the aperture and compressing it into a single channel. In this single-pixel paradigm, each measurement at the RF port corresponds to the projection of the far-field source incident at ($\theta ,\varphi$), $P = e^{{ - jk_{0} \left( {x\sin \theta \cos \varphi + y\sin \theta \sin \varphi } \right)}}$, onto a given modulation pattern across the aperture. As a result, the far-field source can be formed as a linear superposition of the patterns weighted by the respective measurements (*g*_*m*_(*ω*)), where *m* is the *m*th distinct sequence of random *on* and *off* states defined as a *tuning state* and *ω* is the angular frequency. For instance, the programmable aperture can multiplex spatial and spectral information onto a series of sequential single-port measurements by randomly tuning the states of switches. Afterwards, in the computational layer, advanced polarimetric reconstruction techniques involving the pseudo-inversion of the linear matrix equation describing the system forward model, such as compressive single-pixel^30^ or spectral imaging^31^, can be used to retrieve the source information angle and polarization from the single-port compressed measurements. Defining a 2D plane capturing the radiated fields from the wave-chaotic aperture in the near-field (i.e. characterization plane), an estimate of the source projection patterns on the characterization plane $P_{est} = \left[ {P_{{x_{est} }} ,P_{{y_{est} }} } \right]$ corresponding to *x*- and *y*-polarizations of the incoming wave can be retrieved from the compressed measurement as follows:

1 $$ P_{{x_{est} }} = E_{x}^{\dag } g $$

2 $$ P_{{y_{est} }} = E_{y}^{\dag } g $$

where .^†^ is the conjugate transpose operator and *E* = [*E*_*x*_, *E*_*y*_] is the projection of the aperture radiated fields, in *x*- and *y*-polarization states, on a characterization plane calculated by:

3 $$ E_{m} \left( {\overline{r},\omega } \right) = \mathop \smallint \limits_{{r^{\prime}}} m_{m} \left( {\overline{r}^{^{\prime}} ,\omega } \right)G\left( {\overline{r},\overline{r}^{^{\prime}} ,\omega } \right)d\overline{r}^{^{\prime}} $$

where $\overline{r}^{^{\prime}}$ is the coordinates of the characterization plane, $\overline{r}$ is the equivalent aperture plane, $m_{m} \left( {\overline{r}^{^{\prime}} ,\omega } \right)$ is the discrete dipole moments on the antenna aperture, *p* = *x*, *y* denotes the polarization of the field element along *x* and *y* axis while $G\left( {\overline{r},\overline{r}^{^{\prime}} ,\omega } \right)$ is the Green’s function defined as

4 $$ G\left( {\overline{r}_{1} ,\overline{r}_{2} ,\omega } \right) = \frac{{e^{{ - jk\left| {\overline{r}_{2} - \overline{r}_{1} } \right|}} }}{{\left| {\overline{r}_{2} - \overline{r}_{1} } \right|}} $$

Once an estimate of the far-field source on the characterization plane is retrieved from the compressed measurements (*g*) in Eqs. (1) and (2), taking the Fourier transform of *P*_est_ retrieves the DoA information.

### Design of dual-polarized metamaterial element

**Figure 2:**
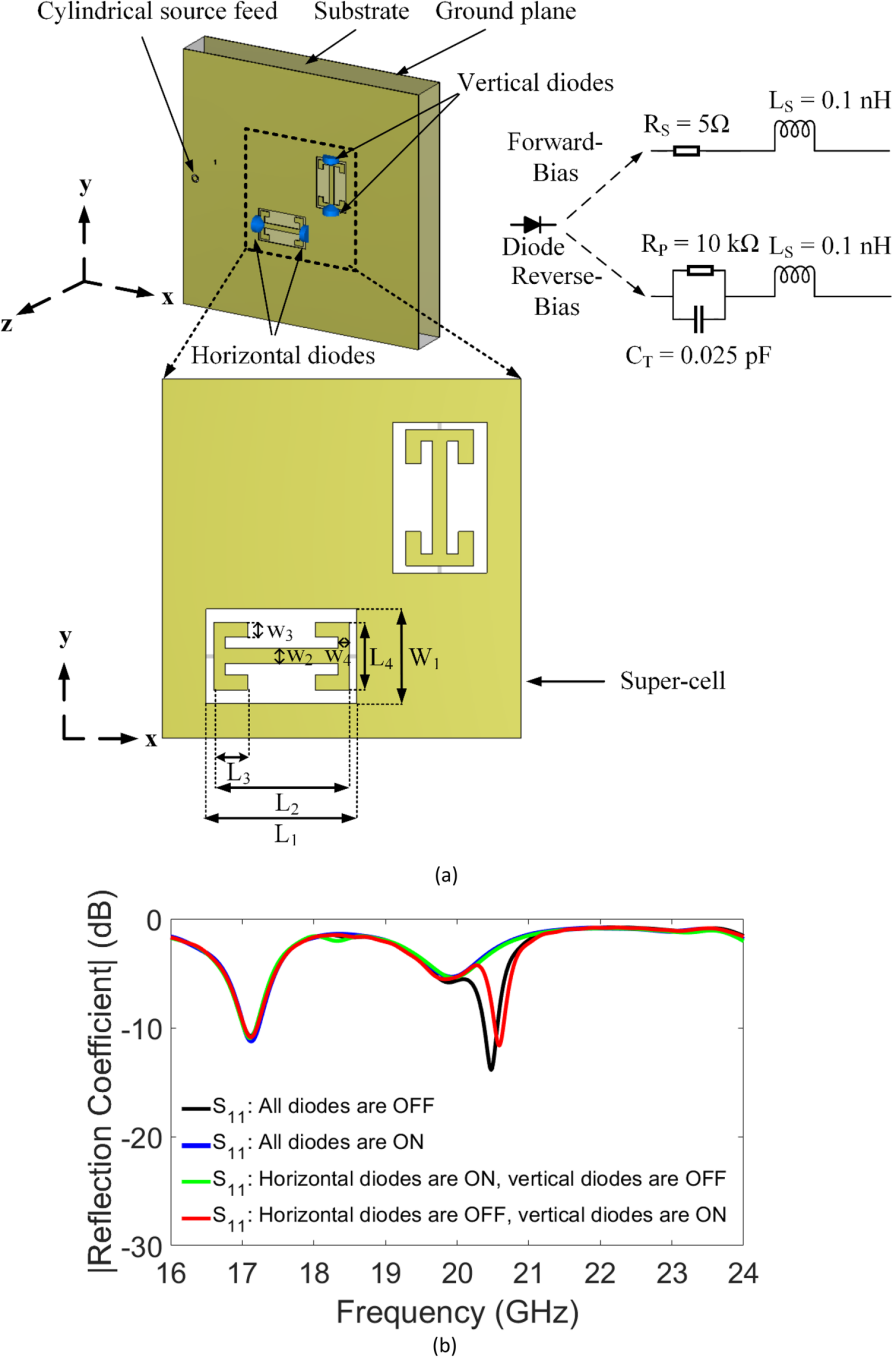
(**a**) Full-wave model of the proposed super-cell with diodes (blue), (**b**) Simulated reflection-coefficient of the tunable super-cell at *on* and *off* states.

Metasurfaces and metasurface-based antennas have been shown for manipulating the wavefronts of anomalous reflection and refraction waves^32–35^, controlling the polarization of waves^36,37^, wireless applications^38–40^ and implementing computational imaging schemes^41,42^. Especially, the recent coded-aperture-based metasurfaces, which can achieve dynamically controllable functionalities by programmability, set up a bridge between the digital and physical worlds^43–45^. In this work, a metamaterial radiator element is chosen as the dual-polarized radiator element to build a polarimetric metasurface aperture for the purpose of polarimetric DoA estimation. Here, we propose a switchable metamaterial radiator shown in Fig. 2a, integrated with four PIN diodes. First, the metamaterial radiator with two orthogonally separated complementary electric inductive-capacitive (cELC) elements is designed as a super-cell structure to radiate and capture both cross- and co-polarized components efficiently. The proposed metamaterial element brings the main property of the cELC resonator^46^ of only responding to in-plane magnetic field. Then, four diodes are introduced across the capacitive gaps in orthogonal positions to achieve polarization state selectivity in the cELC elements. PIN diodes are conductive in the forward bias state, and capacitive in the reverse bias state. When the diodes are in *on* state, they exhibit a low impedance (ideally short-circuit), short-circuiting the cELC elements, thus the metamaterial elements are *off*, i.e., weakly radiating. In contrast, when the diodes are in *off* state, they exhibit a high impedance (ideally open-circuit), preserving the electrical characteristics of the cELC elements, thus the metamaterial elements are *on*, i.e., radiating. The diodes are sequentially switched *off* (reverse-biased) or *on* (forward-biased). The PIN diodes are modeled as a series inductor-resistor circuit in their conducting state (forward-bias) and as an inductor in series with a parallel capacitor-resistor in their non-conducting state (reverse-bias) as depicted in Fig. 2a. The values of the inductance, resistance, and capacitance depend on the diode selection. In this work, MACOM MADP000907-14020 W PIN diode^41,42^ is selected. Both the *on* and *off* states have a package inductance L_S_ = 0.1 nH. The equivalent circuit for the *on* state (forward-biased) has a low resistance R_S_ = 5 Ω which contributes to the insertion loss. The equivalent circuit for the *off* state (reverse-biased) has a parallel combination of the parallel reverse bias resistance R_P_ = 10 kΩ and the total capacitance C_T_ = 0.025 pF contributing to the isolation at the operating frequency of 21 GHz. The dimensions of the proposed metamaterial element are L_1_ = 2 mm, L_2_ = 1.8 mm, L_3_ = 0.4 mm, L_4_ = 0.9 mm, W_1_ = 1.25 mm, W_2_ = W_3_ = 0.2 mm, W_4_ = 0.15 mm. The lower portion of the K-band of 20–22 GHz is selected to be the operating frequency band in this work. Therefore, each metamaterial element must be resonant within K-band to form the coded metasurface aperture operating at these frequencies. In addition, the metamaterial elements should, when in the *on* state, guarantee that ample energy is radiated onto the scene. Figure 2a shows the setup used to analyze the super-cell design in the CST Microwave Studio simulation. The simulated |S_11_| in Fig. 2b depicts the resonant frequency of the element designed to be between 20 and 22 GHz, covering the lower portion of the K-band as a function of different diode states loading the cELC elements within the super-cell structure.

**Figure 3:**
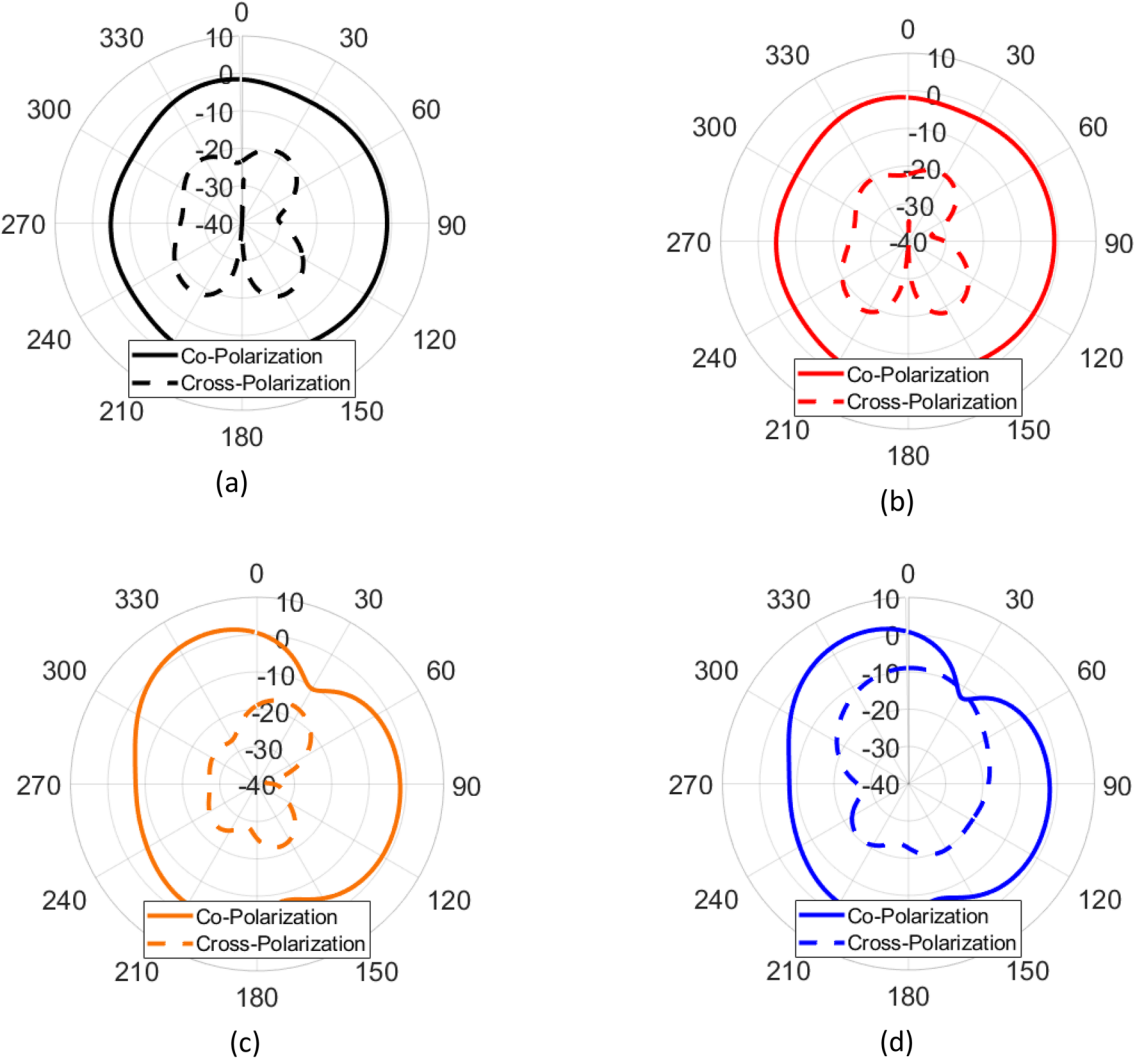
Radiation patterns of the super-cell structure as a function of diode configurations when (**a**) all diodes are *on*, (**b**) horizontal diodes are *on* and vertical diodes are *off*, (**c**) horizontal diodes are *off* and vertical diodes are *on,* and (**d**) all diodes are *off*. Unit: dBi.

The radiation patterns of the super-cell as a function of diode configurations are shown in Fig. 3. It is observed that the radiation from the element is strong with low cross-polarization levels for all diode states as shown in Fig. 3. When all diodes are *on*, or the horizontal diodes are switched *off* and the vertical state is switched *on*, the radiation patterns confirm that the structure radiates weakly into either the co- or cross-pol states. The simultaneously switched *on* or *off* configuration of the diodes is selected for DoA estimation as described in the next section.

**Figure 4:**
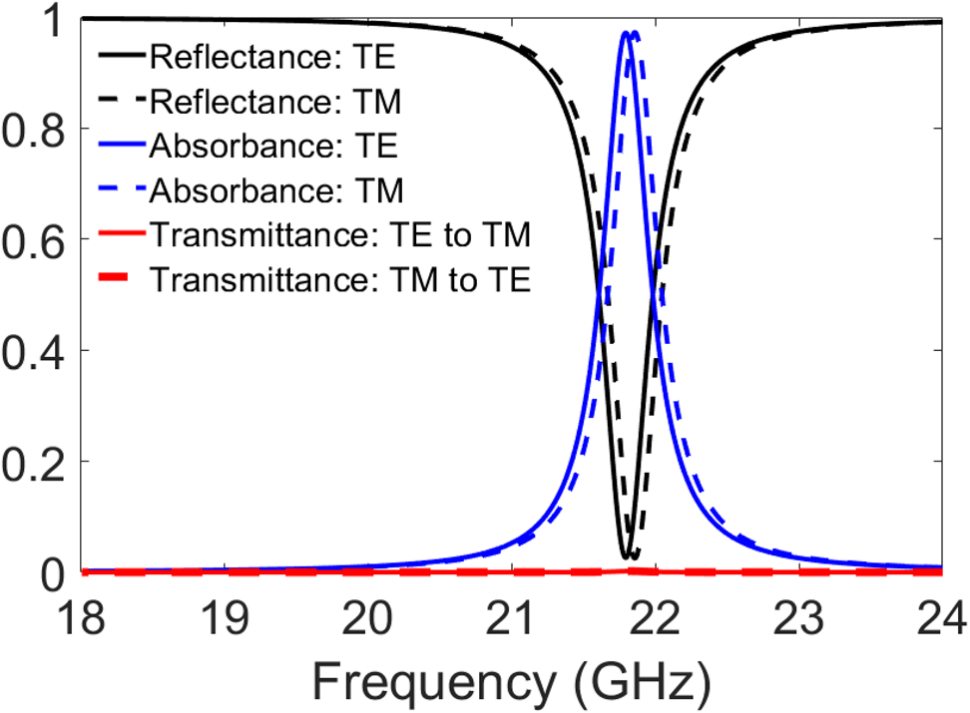
Reflectance, transmittance, and absorbance of the proposed super-cell for normal incidence.

Next, we demonstrate the sensitivity of the structure to the polarization state of the incoming wave. A linearly polarized plane wave can be a transverse electric (TE)-polarized wave or a transverse magnetic (TM)-polarized wave. Therefore, the reflectance, transmittance, and absorbance spectra corresponding to the super-cell need to be investigated with regards to the polarization of the incident wave. Figure 4 shows the reflectance, transmittance, and absorbance of the proposed super-cell for normal incidence. It is observed that the element has a high absorbance of around 97% at the operating frequency. This demonstrates that the power of the incident wave is absorbed thoroughly with negligible specular reflection or scattering. Furthermore, the transmittance from TE mode to TM mode, and vice versa, is insignificant. The low coupling between two modes guarantees that the structure distinguishes the polarization state of the incident wave clearly and efficiently.

### Design of programmable metasurface aperture

**Figure 5:**
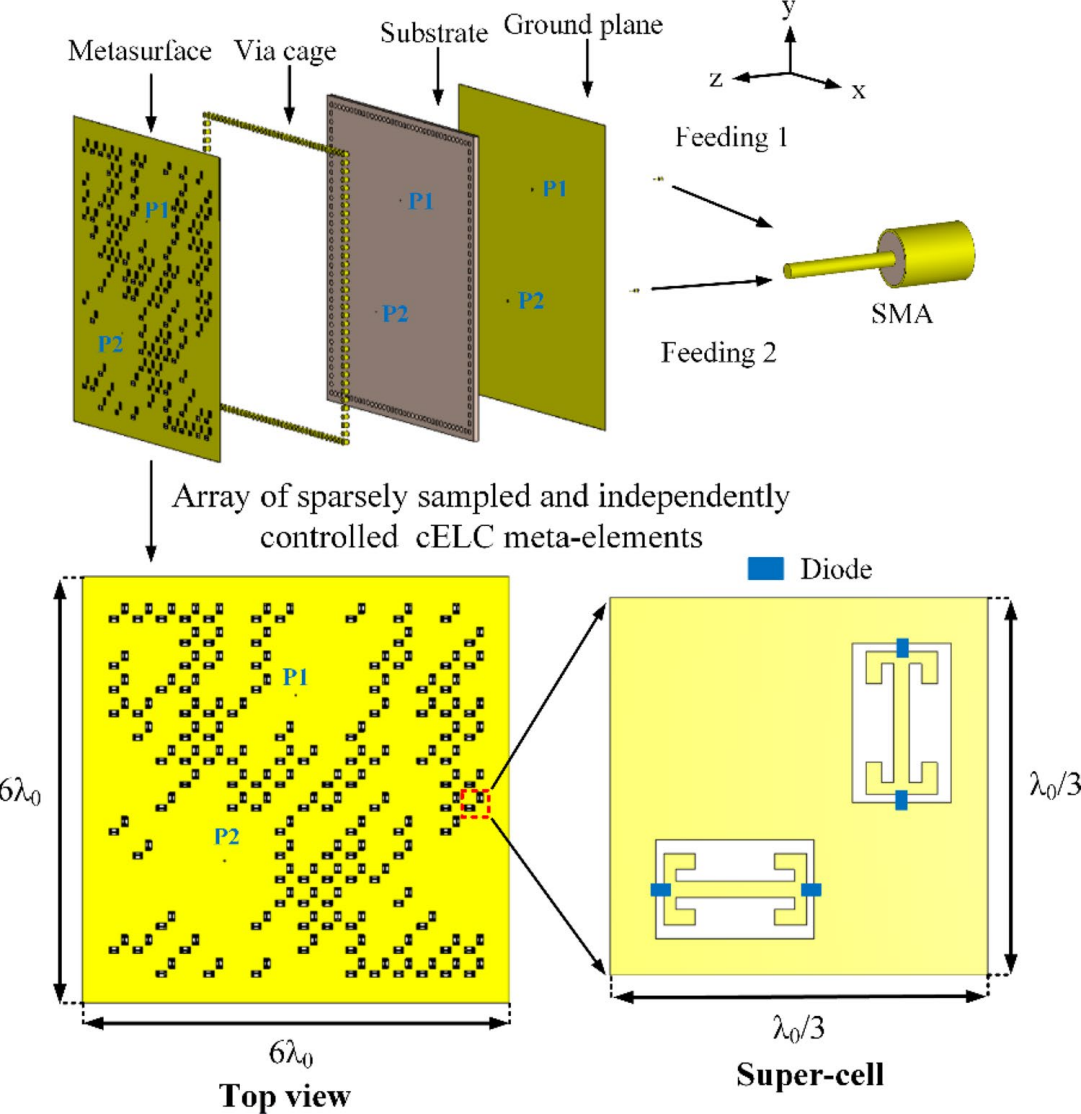
Diagram of two-port programmable metasurface aperture using an array of sparsely sampled and independently controlled cELC meta-elements.

The programmable metasurface aperture used in this work is a printed cavity formed by a double-sided copper cladding dielectric substrate bounded by a metallic via fence, as depicted in Fig. 5. The metal walls (facilitated by the via fence) and the top and bottom copper layers form the boundaries of the cavity volume composed of a 1.52-mm thick Rogers RO4003C dielectric substrate (ε_r_ = 3.35, tan δ = 0.0027). Two RF coaxial connectors are connected to the cavity, launching a cylindrical wavefront inside the cavity structure. The upper conductive layer is fashioned with a 2D array of sparse two orthogonally separated cELC radiator metamaterial elements, with the resonant frequencies distributed between 20 and 22 GHz. The complex fields formed within the cavity radiate into free-space by superposing the contributions from each of the radiating metamaterial elements. By dynamically switching the states of the diodes, programmable manipulation of the radiation patterns is achieved. In this context, each metasurface aperture configuration with a specific pattern of *on* and *off* diodes is called a *mask*, producing a radiation pattern that changes as the mask is varied, i.e. spatio-temporal variation. These masks allow the projection of the incoming far-field source patterns to be probed using a number of spatio-temporally incoherent field patterns radiated from the programmable metasurface as a function of varying mask configurations. The size of the electrically large cavity is *6λ*_0_ × *6λ*_0_, where *λ*_0_ is the free-space wavelength at 21 GHz, allowing the metasurface to replace a large number of antennas. To put this statement into context, synthesizing the same aperture, *6λ*_0_ × *6λ*_0_, at the Nyquist limit (*λ*_0_/2) would require 13 × 13 individual antennas, and hence data acquisition channels, whereas the 2D programmable metasurface has only one channel.

**Figure 6:**
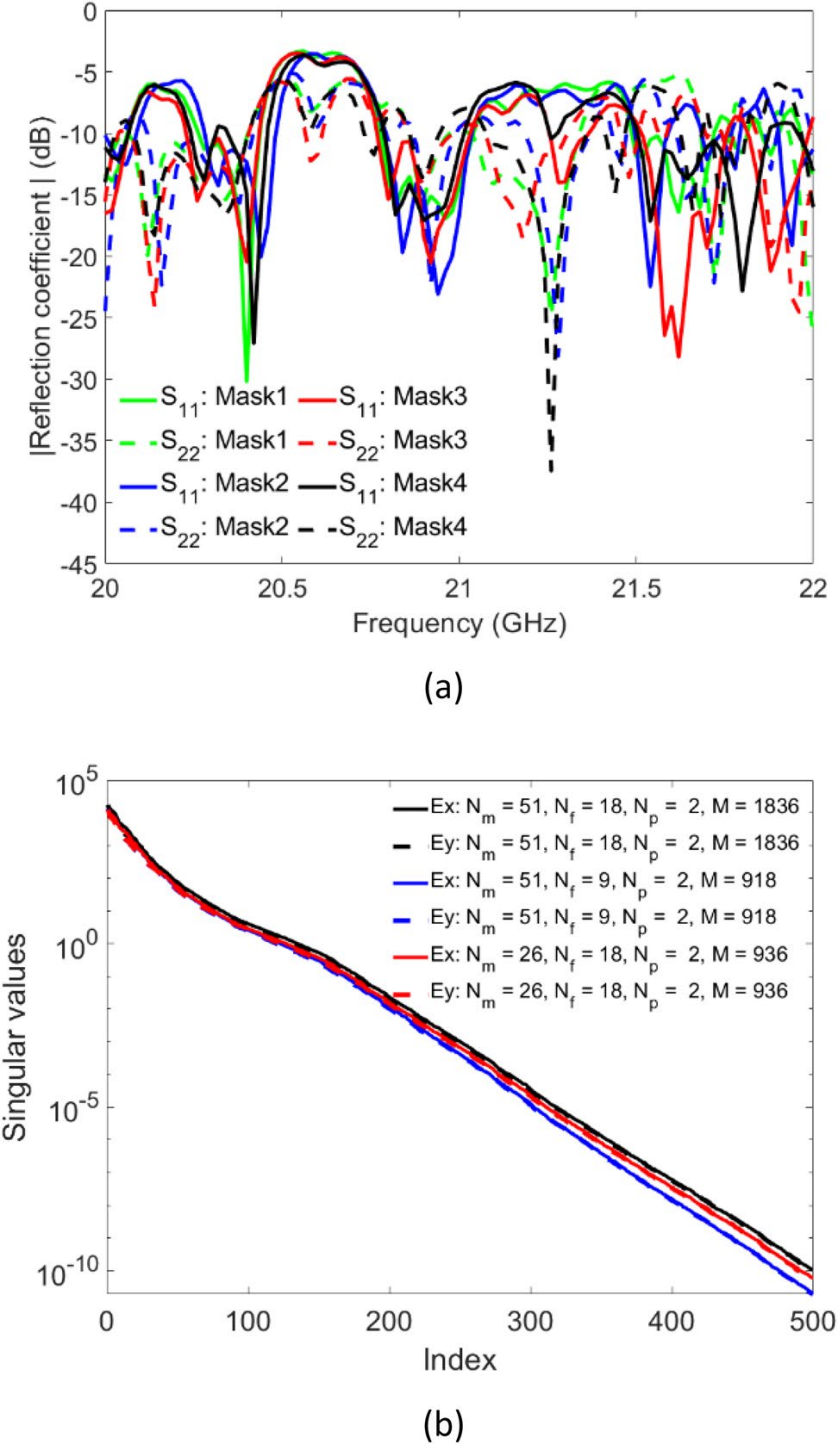
Simulated (**a**) reflection coefficient at each waveguide port and (**b**) singular value decomposition.

The simulated reflection coefficient patterns of the proposed metasurface aperture are shown in Fig. 6a. Analyzing Fig. 6a, the average reflection coefficients of the metasurface remain around − 10 dB, ensuring that the wavefront launched into the cavity structure is effectively radiated into free-space through the metamaterial elements and the signal reflected back to the feeding port is not significant. As expected, we also observe a significant variation in the reflection coefficient pattern as a function of tuning states (i.e. each mask state) within the frequency band of 20–22 GHz. A multitude of resonances represented by the dips in the reflection coefficient pattern corresponding to the variation of the cavity modes can be observed for all four masks. Because the modes are reconfigured, the elements are excited by new fields for each of these masks. In this context, it is worth mentioning that the states of the diodes can be dynamically tuned, i.e. the diodes can perform not only amplitude modulation but also phase modulation^47^. However, only the amplitude modulation is investigated in this work.

**Figure 7:**
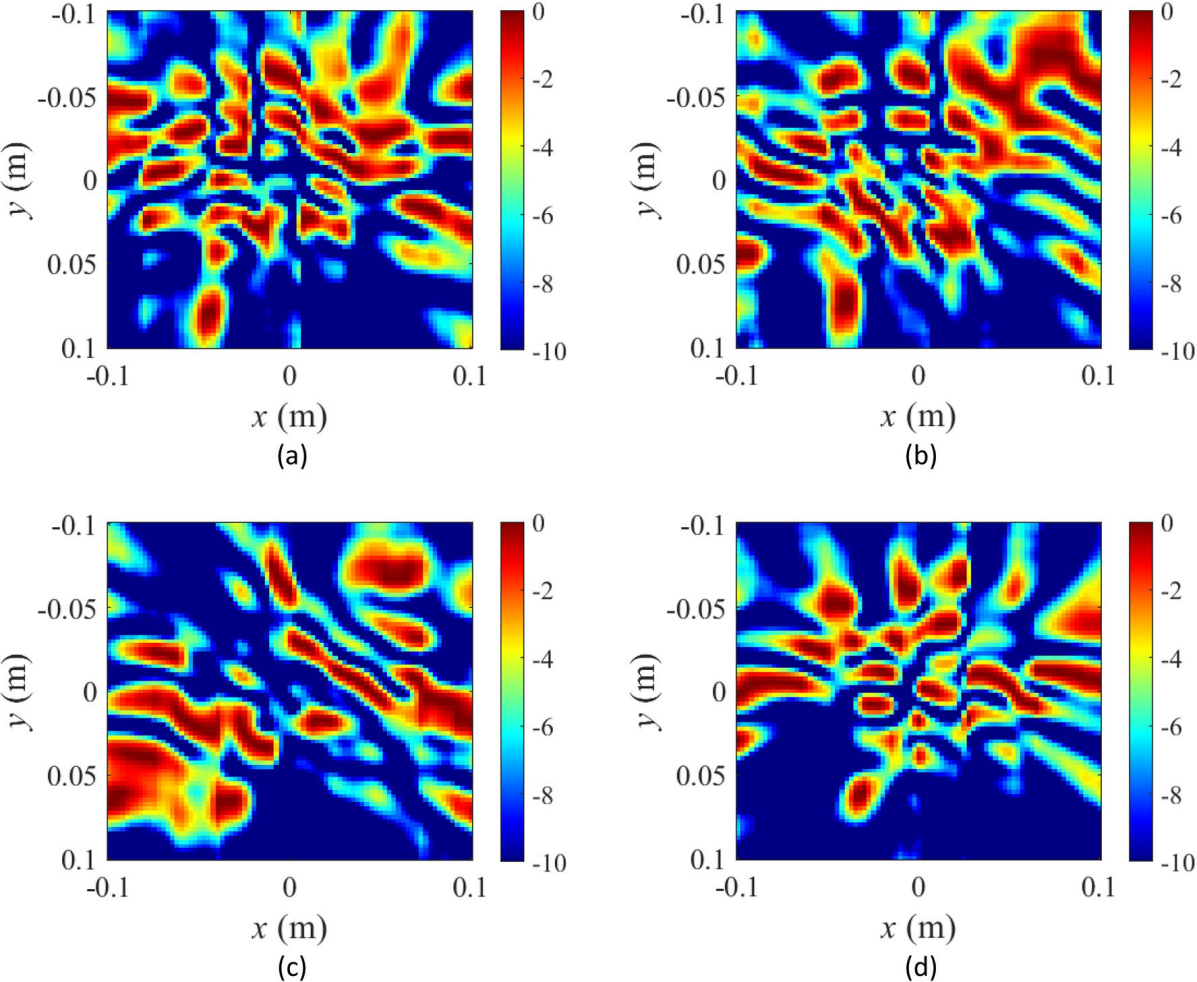
Simulated near-field patterns of the electric-field radiated by the proposed metasurface aperture (**a**) mask 1, (**b**) mask 2, (**c**) mask 3, and (**d**) mask 4. Frequency of operation is 21 GHz. Unit: dB.

Moreover, singular value decomposition (SVD) is used to evaluate the orthogonality of the measurement modes^48^ which is indicative of the correlation among the fields radiated from the metasurface. SVD results are presented in Fig. 6b. It is observed that as the number of masks or the number of measurements is increased, the slope of the SVD pattern becomes flatter. The purpose of using the two RF ports is to increase the number of measurements without increasing the frequency points and the tuning state of the diodes. In Fig. 7, the diversity of the near-field patterns as a function of varying masks can be appreciated, which is a key component for the wave-chaotic operation that is necessary to encode the radar measurements using programmable metasurface apertures^14,49^. This implies that the radiation patterns need to change dynamically by tuning the masks, and that the radiated modes are orthogonal. The spatial, quasi-random variation of the field patterns radiated by the metasurface, as depicted in Fig. 7, ensures that the spectrum of far-field wavefronts that are incident on the metasurface aperture can be probed using the compressive sensing theory to retrieve DoA information. This wave-chaotic operation is key as it replaces the conventional, sequential raster-scan based sampling techniques, which, typically rely on multi-pixel receiver hardware.

## Results and discussion

In this work, we selected the size of the metasurface aperture as *D* = 85.68 mm. We first extract the radiated fields from the metasurface aperture in CST Microwave Studio. Once the radiated fields from the metasurface are obtained, we can calculate the field distribution at any other given distance using Green’s functions^50^. The number of frequency points is selected to be 51 in the frequency range of 20–22 GHz, while the number of masks is selected to be 18 and the number of ports is 2. Hence, the number of wave-chaotic modes (or number of measurements) generated by the polarimetric metasurface is equal to 1836.

**Figure 8:**
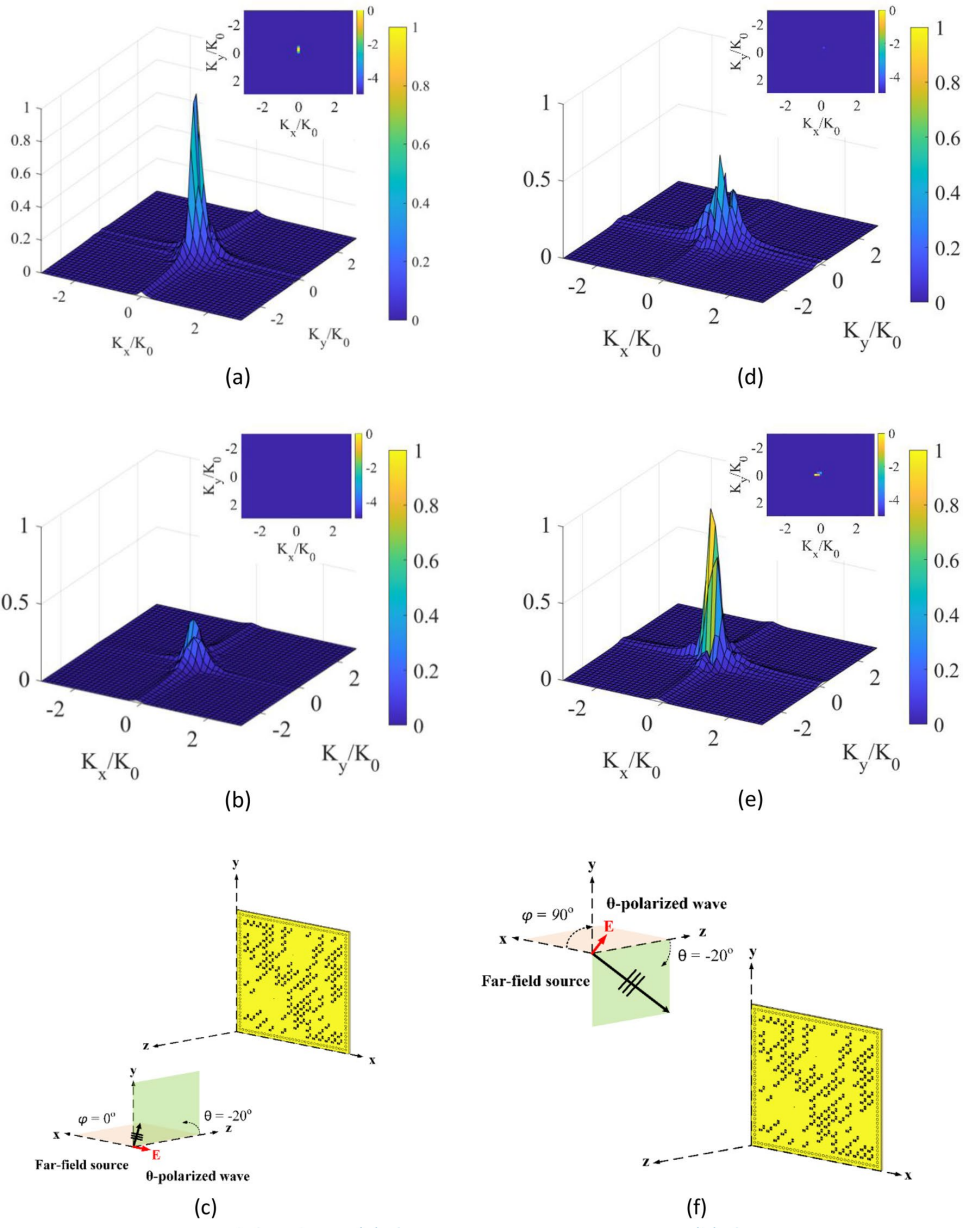
DoA estimation at left column (**a**) $\theta_{x}$ reconstructed DoA pattern, (**b**) $\theta_{y}$ reconstructed DoA pattern at a $\theta$-polarized incident of $\theta = - 20^\circ$ and $\varphi = 0^\circ$ with normalized magnitudes, and (**c**) depiction of the far-field incident wave on the polarimetric metasurface aperture. DoA estimation at right column (**d**) $\theta_{x}$ reconstructed DoA pattern, (**e**) $\theta_{y}$ reconstructed DoA pattern at a $\theta$-polarized incident of $\theta = - 20^\circ$ and $\varphi = 90^\circ$ with normalized magnitudes, and (**f**) depiction of the far-field incident wave on the polarimetric metasurface aperture. 3D DoA pattern is in linear scale, and 2D DoA pattern on top is in dB scale.

First, we start with the DoA estimation scenario consisting of a single source placed in the far-field of the radar aperture. The aperture is synthesized using the developed programmable polarimetric metasurface depicted in Fig. 5. For this analysis, a $$\theta$$-polarized wave with an incident angle of $$\theta = - 20^\circ$$ and $$\varphi = 0^\circ$$ is selected. In the $$\theta$$-polarized incident wave scenario, the direction of the E-field is along the unit vector of $$\theta$$, represented in a polar coordinate system. In this case, the incident wave is a TM wave. After taking the 2D Fourier transform of the retrieved source projections from Eqs. (1) and (2), we obtain the DoA patterns shown in Fig. 8a–c. It is observed that the retrieved source projection pattern in $$x$$-polarization ($$P_{{x_{est} }}$$), named here as “$$\theta_{x}$$ reconstructed DoA pattern”, is dominant compared with the retrieved source projection pattern in y-polarization ($$P_{{y_{est} }}$$), named here as “$$\theta_{y} $$ reconstructed DoA pattern”. The reason is that the direction of the E-field of the incident wave is along the $$x$$-axis, therefore, the incident wave couples only with the $$x$$-polarized component of the metasurface aperture. Next, the polarization of the incident wave is fixed at $$\theta$$-polarization, but the angle of the source is changed to $$\theta = - 20^\circ $$ and $$\varphi = 90^\circ$$. The reconstructed DoA patterns are shown in Fig. 8d–f. In this case, the $$\theta_{y} $$ reconstructed DoA pattern is dominant when compared with the $$\theta_{x} $$ reconstructed DoA pattern. Because the direction of the E-field along in $$y$$–$$z $$ plane, the incident wave couples only with the y-polarized component of the metasurface aperture. From these results, it is evident that the developed single-pixel DoA estimation concept can distinguish the polarization state of the incident source waveform.

**Figure 9:**
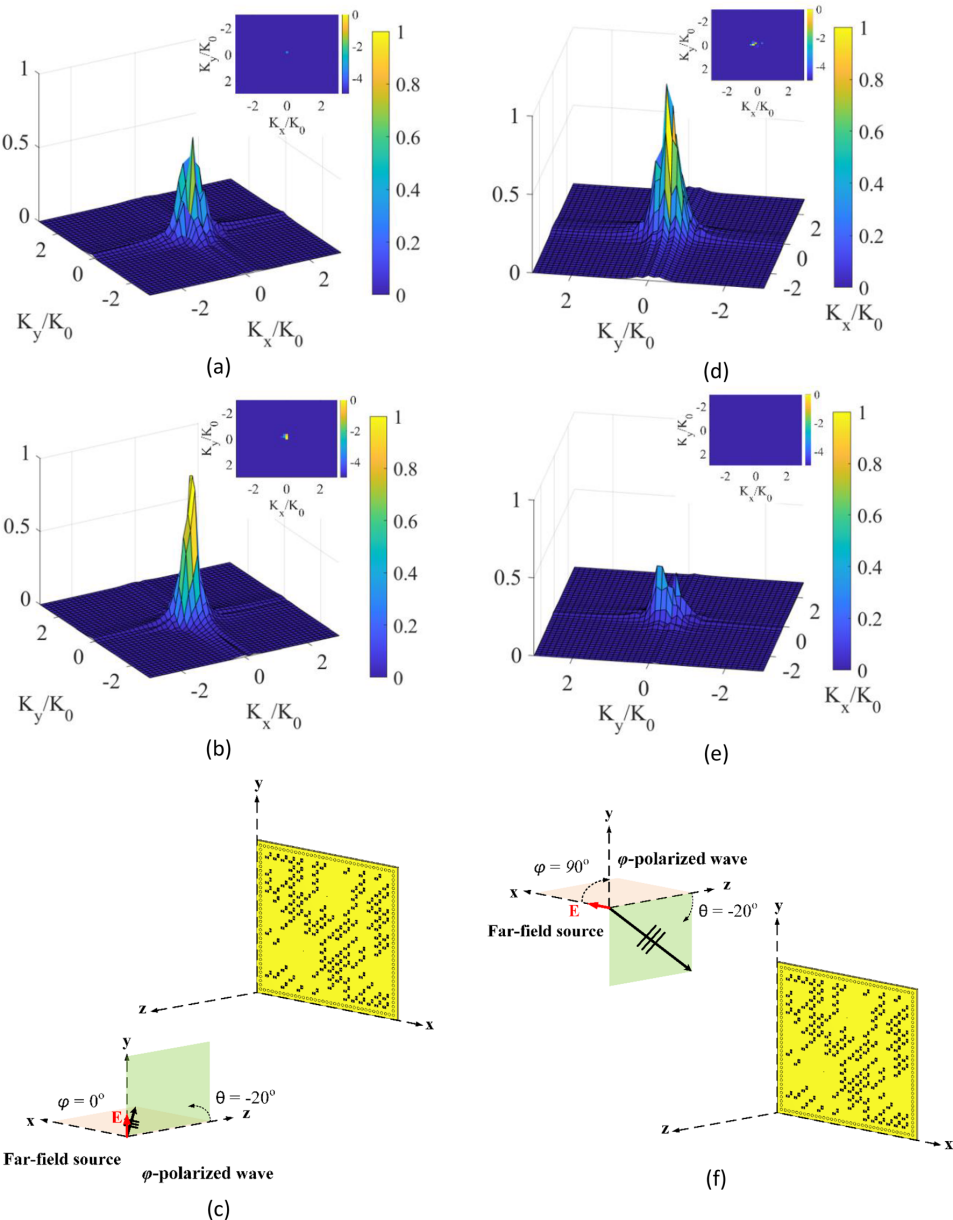
DoA estimation at left column (**a**) $\varphi_{x}$ reconstructed DoA pattern, (**b**) $\varphi_{y}$ reconstructed DoA pattern at a $\varphi$-polarized incident of $\theta = - 20^\circ$ and $\varphi = 0^\circ$ with normalized magnitudes, and (**c**) depiction of the far-field incident wave on the polarimetric metasurface aperture. DoA estimation at right column (**d**) $\varphi_{x}$ reconstructed DoA pattern, (**e**) $\varphi_{y}$ reconstructed DoA pattern at a $\varphi$-polarized incident of $\theta = - 20^\circ$ and $\varphi = 90^\circ$ with normalized magnitudes, and (**f**) depiction of the far-field incident wave on the polarimetric metasurface aperture. 3D DoA pattern is in linear scale, and 2D DoA pattern on top is in dB scale.

Second, the polarization of the incident wave is changed to a $\varphi$-polarized incident wave. In the $\varphi$-polarized incident wave scenario, the direction of the E-field is along the unit vector of $\varphi$ in polar coordinates. In this case, the incident wave is a TE wave. Figure 9a–c show the reconstructed DoA patterns when the incident angle of the source is $\theta = - 20^\circ$ and $\varphi = 0^\circ$. In this case, because the direction of the E-field is along the $y$-axis, the $\varphi_{y}$ reconstructed DoA pattern becomes dominant. In contrast, when the incident angle of the $\varphi$-polarized source is set to be $\theta = - 20^\circ$ and $\varphi = 90^\circ$, the $\varphi_{x}$ reconstructed DoA pattern becomes dominant, as shown in Fig. 9d–f.

**Figure 10:**
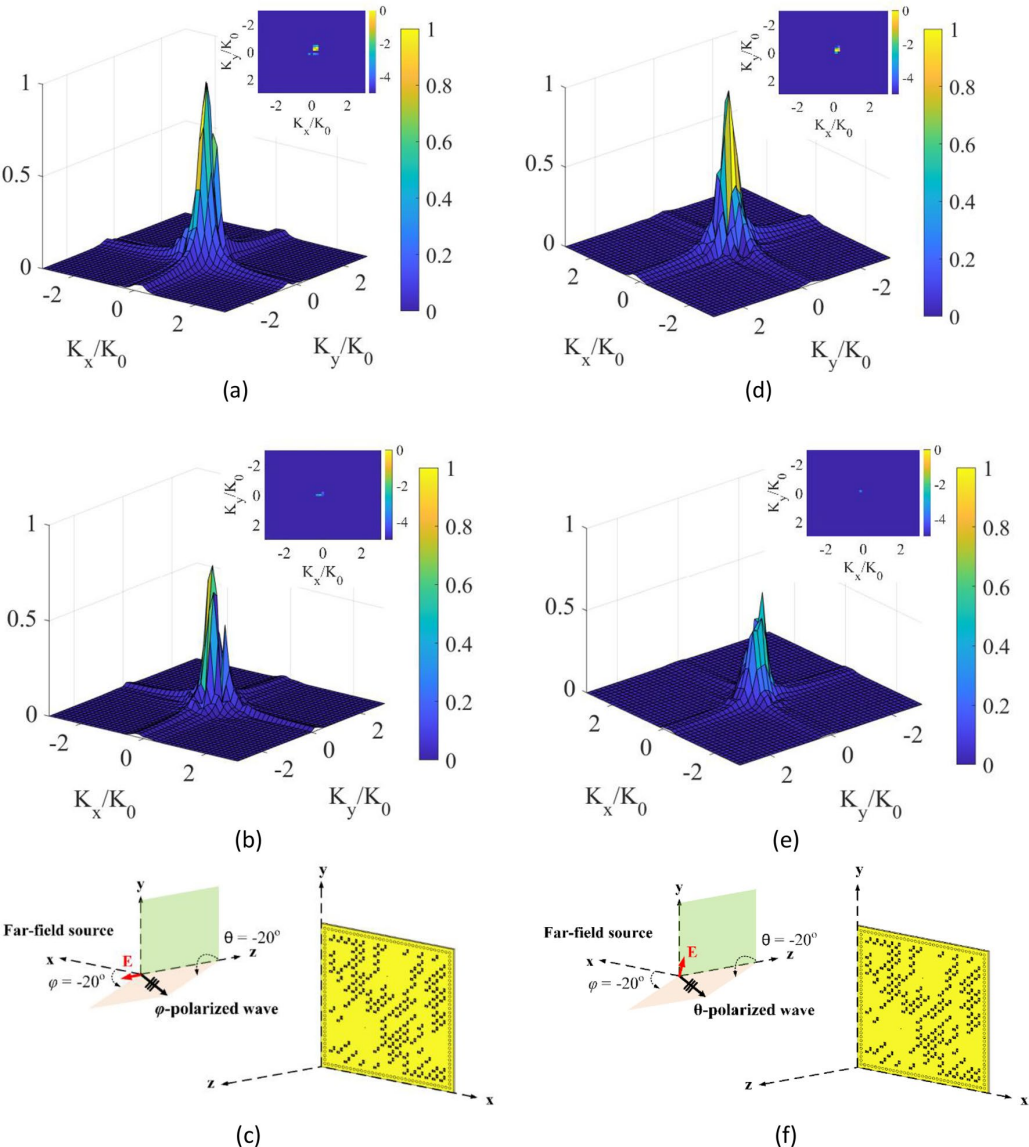
DoA estimation at left column (**a**) $\varphi_{x}$ reconstructed DoA pattern, (**b**) $\varphi_{y }$ reconstructed DoA pattern at a $\varphi$-polarized incident of $\theta = - 20^\circ$ and $\varphi = - 20^\circ$ with normalized magnitudes, and (**c**) depiction of the far-field incident wave on the polarimetric metasurface aperture. DoA estimation at right column (**d**) $\theta$-$x$ reconstructed DoA pattern, (**e**) $\theta$-$y$ reconstructed DoA pattern at a θ-polarized incident of $\theta = - 20^\circ$ and $\varphi = - 20^\circ$ with normalized magnitudes, and (**f**) depiction of the far-field incident wave on the polarimetric metasurface aperture. 3D DoA pattern is in linear scale, and 2D DoA pattern on top is in dB scale.

Third, the incident angle of the source is changed to $\theta = - 20^\circ$ and $\varphi = - 20^\circ$. In this case, for both $\varphi$- and $\theta$-polarized incident waves, the retrieved source projection pattern in $x$-polarization is higher in intensity, as shown in Fig. 10. This proves that the sensitivity of the structure to the x- polarization state is higher than that of the y-polarization state.

**Figure 11:**
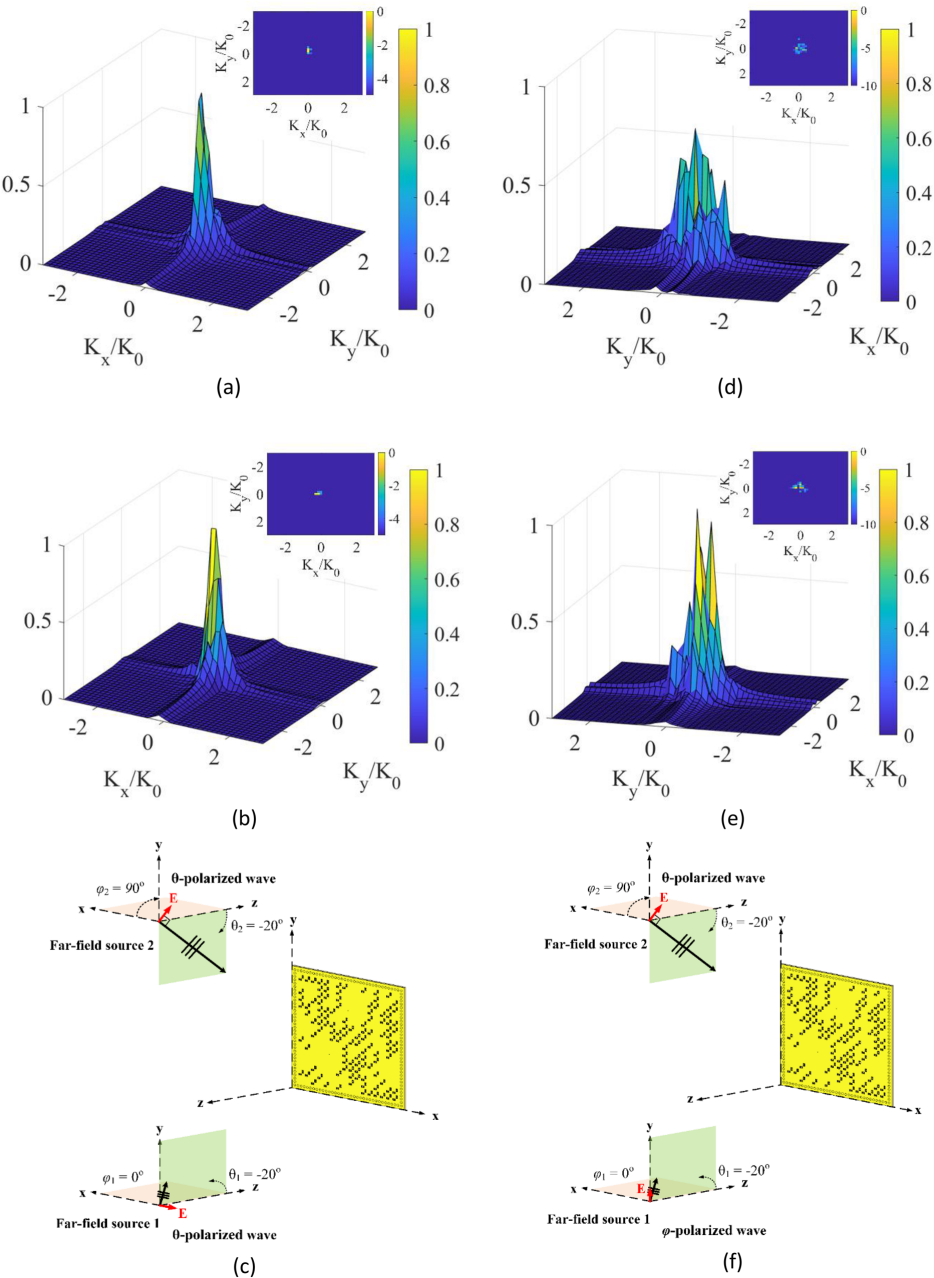
DoA estimation at left column (**a**) $\theta_{x}$ reconstructed DoA pattern, (**b**) $\theta_{y}$ reconstructed DoA pattern at a $\theta$-polarized incident of $\theta$_1_ = − 20° and $\varphi_{1} = 0^\circ$ and a θ-polarized incident of $\theta$_2_ = − 20° and $\varphi_{2} = 90^\circ$ with normalized magnitudes, and (**c**) depiction of the far-field incident waves on the polarimetric metasurface aperture. DoA estimation at right column (**d**) $x$-polarization reconstructed DoA pattern, (**e**) $y$-polarization reconstructed DoA pattern at a $\varphi$-polarized incident of $\theta_{1} = - 20^\circ$ and $\varphi_{1} = 0^\circ$ and a $\theta$-polarized incident of $\theta_{2} = - 20^\circ$ and $\varphi_{2} = 90^\circ$ with normalized magnitudes, and (**f**) depiction of the far-field incident wave on the polarimetric metasurface aperture. 3D DoA pattern is in linear scale, and 2D DoA pattern on top is in dB scale.

Next, we demonstrate the ability of the proposed metasurface aperture in finding the direction for multiple sources while also retrieving the polarization states. To achieve this, two sources illuminating the aperture at ($\theta_{1} = - 20^\circ$, $\varphi_{1} = 0^\circ$) and ($\theta_{2} = - 20^\circ$, $\varphi_{2} = 90^\circ$) incident angles with the same polarization and different polarization are investigated. For this analysis, first, a $\theta$-polarized incident of $\theta_{1} = - 20^\circ$ and $\varphi_{1} = 0^\circ$ and a $\theta$-polarized incident of $\theta_{2} = - 20^\circ$ and $\varphi_{2} = 90^\circ$ is selected. Two incident waves couple with the two polarization components of the metasurface aperture. Therefore, we can clearly observe the $\varphi_{x}$ and $\varphi_{y}$ reconstructed DoA patterns in Fig. 11a–c. The $\varphi_{x}$ reconstructed DoA pattern is caused by $\theta$-polarized incident of $\theta_{2} = - 20^\circ$ and $\varphi_{2} = 90^\circ$, while the $\varphi_{y }$ reconstructed DoA pattern is caused by $\theta$-polarized incident of $\theta_{1} = - 20^\circ$ and $\varphi_{1} = 0^\circ$. Moreover, a $\varphi$-polarized incident of $\theta_{1} = - 20^\circ$ and $\varphi_{1} = 0^\circ$ and a $\theta$-polarized incident of $\theta_{2} = - 20^\circ$ and $\varphi_{2} = 90^\circ$ illuminates the aperture. As shown in Fig. 11d–f, the $y$-polarization reconstructed DoA pattern clearly depicts two peaks. This is because both incident waves couple with the $y$-polarized component of the metasurface aperture. The results show that the proposed dynamically programmable DoA technique can achieve direction finding for multiple sources with the same polarization or with different polarization components. More enhanced reconstructions and resolutions can be achieved by increasing the number of modes and/or using a larger aperture at an expense of increased computational and hardware complexity.

**Figure 12:**
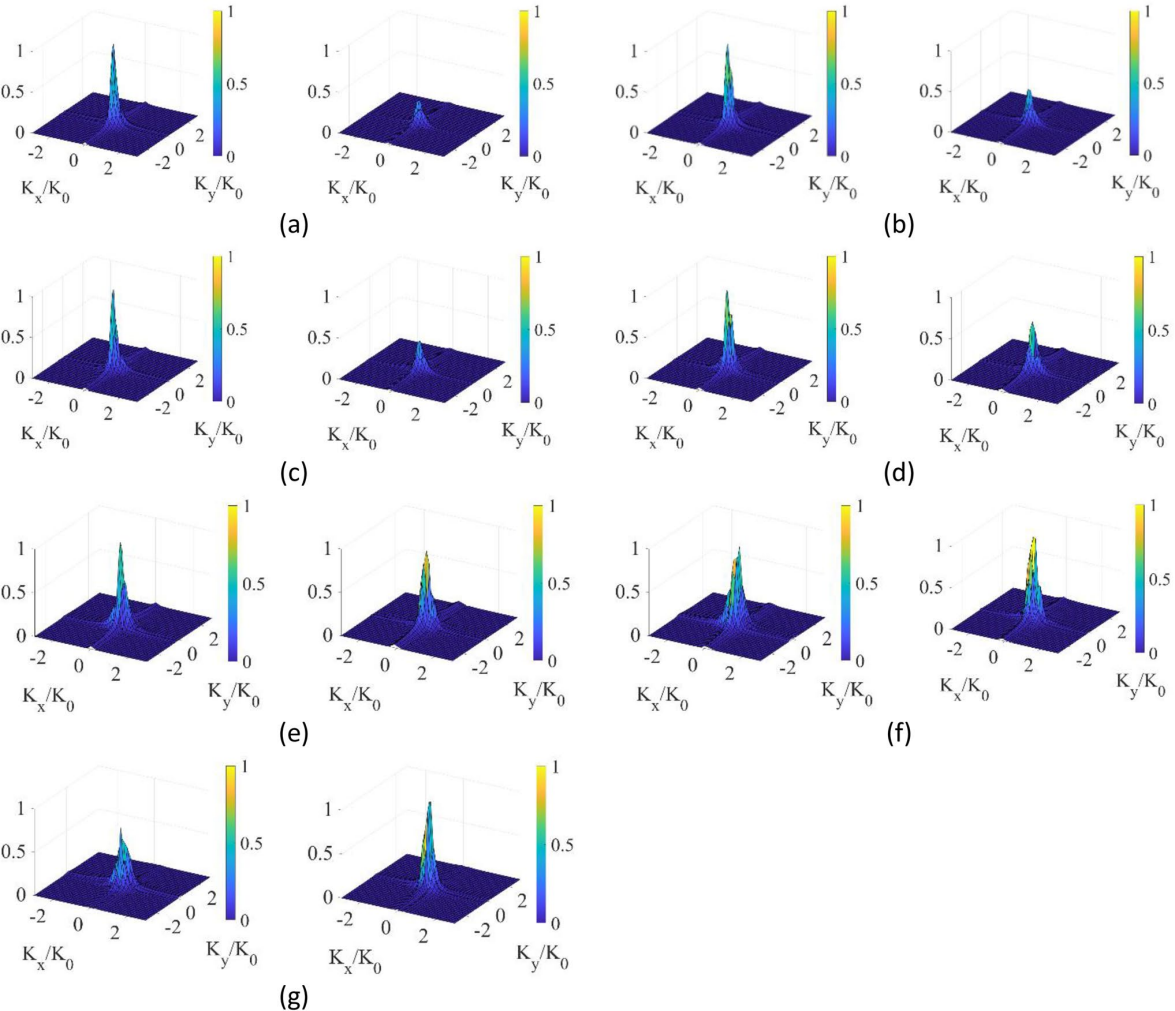
DoA estimation $x$-polarization reconstructed DoA pattern on the left columns and $y$-polarization reconstructed DoA pattern on the right columns at a $\theta$-polarized incident of $\theta = - 20^\circ$ and $\varphi = 0^\circ$ when E vector is rotated at (**a**) 0°, (**b**) 15°, (**c**) 30°, (**d**) 45°, (**e**) 60°, (**f**) 75°, and (**g**) 90° with normalized magnitudes.

**Figure 13:**
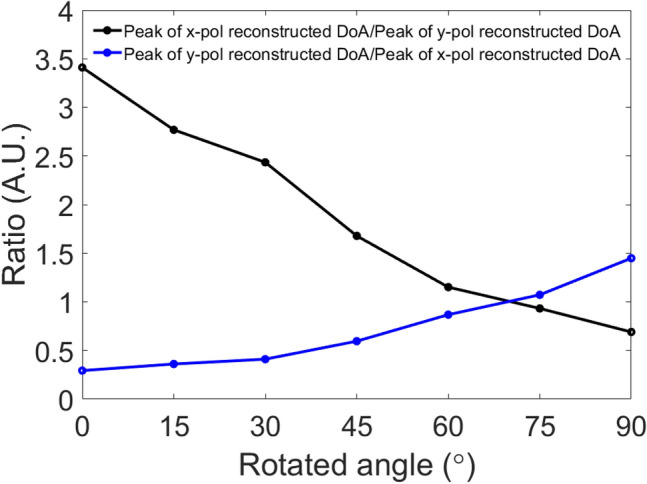
Ratio of peak of $x$-polarization reconstructed DoA spectrum to peak of $y$-polarization reconstructed DoA spectrum (black line), and ratio of peak of $y$-polarization reconstructed DoA spectrum to peak of $x$-polarization reconstructed DoA spectrum (blue line) as a function of the rotated E vector at a $\theta$-polarized incident of $\theta = - 20^\circ$ and $\varphi = 0^\circ$.

Finally, the fixed $\theta$-polarized incident of $\theta = - 20^\circ$ and $\varphi = 0^\circ$ with the rotating E-vector is investigated. $x$-polarization reconstructed DoA patterns and $y$-polarization reconstructed DoA patterns when the E-vector is rotated at 0°, 15°, 30°, 45°, 60°, 75°, and 90° are shown in Fig. 12. Here, E-vector rotated at 0° means that the E-vector is along the $x$-axis and E-vector rotated at 90° indicates that the E-vector is along the $y$-axis. It is observed that the sensitivity of the structure to the $x$-polarization state turns to the $y$-polarization corresponding to changes in the polarization of the incident wave. In Fig. 13, we present the ratio between the peaks of the $x$-polarized and $y$-polarized reconstructed DoA spectrums (black line) and the ratio between the peaks of the $y$-polarized and $x$-polarized reconstructed DoA spectrums (blue line) as a function of the rotated E vector at an $\theta$-polarized incident of $\theta = - 20^\circ$ and $\varphi = 0^\circ$. Figure 13 clearly shows the transition level of the sensitivity of the structure to the change of the polarization of the incident wave.

## Conclusions

In this paper, a polarimetric CS-based radar technique for DoA estimation using a single-pixel dynamically reconfigurable wave-chaotic metasurface antenna, acting as a receiver, was presented. We demonstrated that leveraging the spatio-temporarily incoherent measurement modes generated by the coded programmable metasurface aperture, high fidelity DoA patterns for sources incident in different polarization bases can be retrieved. The results also show that the proposed dynamically programmable DoA technique can achieve direction finding for multiple sources while also retrieving the polarization states. Using this technique, the DoA estimation of far-field sources was shown to require only a single data acquisition channel. This single-pixel compression of the incident source information on the metasurface antenna was achieved through the wave-chaotic transfer function of the programmable metasurface, significantly simplifying the hardware layer in comparison to conventional multi-pixel fully connected antenna array based DoA schemes. Although the programmable metasurface was shown for the K-band frequency regime, the presented technique can readily be adopted at millimeter-wave and submillimeter-wave frequencies for applications requiring superior resolution.
